# Costs associated with adverse drug reactions in an older population admitted to hospital: a prospective cohort study

**DOI:** 10.1007/s00228-023-03552-x

**Published:** 2023-08-24

**Authors:** Kathleen Bennett, Caitriona Cahir, Jan Sorensen

**Affiliations:** 1 Data Science Centre, School of Population Health, RCSI University of Medicine and Health Sciences, Dublin, Ireland; 2 Healthcare Outcomes Research Centre, School of Population Health, RCSI University of Medicine and Health Sciences, Dublin, Ireland

**Keywords:** Adverse drug reaction, Hospitalisation, Healthcare costs, Drug-related side effects

## Abstract

**Purpose:**

This study examines healthcare costs associated with adverse drug reactions (ADR) in an older population admitted acutely to an Irish tertiary hospital.

**Methods:**

Prospective cohort study involving older persons admitted to hospital with and without an ADR. Data was collected at baseline, during hospitalisation and post-discharge. Participants provided information on healthcare resource use three months before admission (baseline) and three months after discharge (follow-up). For each healthcare resource, unit costs were derived and applied. The average cost (standard deviation (SD)) associated with the hospital admission for the ADR and non-ADR are presented. In addition, baseline and follow-up care costs were compared using difference-in-difference analysis and presented with 95% confidence intervals (CI). Costs by preventability and severity of ADR are also presented.

**Results:**

A total of n = 230 participants were included (n = 93 ADR and n = 137 without ADR). The average cost associated with hospital admission for an ADR was €9538 (SD €10442) and €9828 (SD €11770) for non-ADR. The additional follow-up costs (difference-in-difference) associated with the ADR was estimated at €2047 (95% CI: -€889 to €4983). The mean incremental follow-up cost of definite preventable ADRs was estimated at €1648 (95% CI: -€4310 to €7605), possible preventable ADRs €2259 (95 CI: -€1194 to €5712) and unavoidable ADRs €1757 (95% CI: -€3377 to €6890). The mean incremental follow-up cost associated with moderate severe ADRs was estimated at €1922 (95% CI: -€1088 to €4932) and €3580 (95% CI: -€4898 to €12,058) for severe ADRs.

**Conclusion:**

ADRs leading to hospital admission are associated with modest incremental healthcare costs during and three months after admission. Severe and possibly preventable ADRs were associated with higher costs.

**Supplementary Information:**

The online version contains supplementary material available at 10.1007/s00228-023-03552-x.

## Introduction

Exposure to multiple medicines, particularly in older populations with multimorbidity, may cause harm leading to poorer outcomes such as increased risk of death, falls, drug interactions, non-adherence, and hospitalization [1]. Adverse drug reaction (ADR) is one potential type of medication harm which has been defined as ‘an appreciably harmful or unpleasant reaction, resulting from an intervention related to the use of a medicinal product, which predicts hazard from future administration and warrants prevention or specific treatment, or alteration of the dosage regimen, or withdrawal of the product’ [2]. ADRs can result in increased morbidity, hospitalisation and mortality [3, 4].

The economic consequences of ADRs can be considerable with most evidence based on costs associated with hospitalisation and ADRs occurring within the hospital setting [5, 6]. However, there is significant heterogeneity in methodologies and data employed in economic studies of ADRs including differences in the study designs, types of ADRs reported, drugs implicated and types of costs included. Few previous studies have included post-discharge costs and mainly focus on costs associated with hospitalisation for an ADR [5–7]. Despite the limitations of previous studies most have found that the economic cost of ADRs are modest or high which can have significant implications for health services and staff, health payers, and individuals involved [5–7].

Therefore, the aim of this study is to estimate the healthcare costs associated with hospitalisation and post-discharge in an older aged cohort (aged 65 + years) admitted acutely to a tertiary hospital in Ireland with an ADR compared to a similar cohort without an ADR.

## Methods

This study uses the Adverse Drug reactions in an Ageing PopulaTion (ADAPT) cohort [8, 9]. The analysis follows the recommendations issued by the Irish Health Information and Quality Authority (HIQA) [10] and the report follows the STROBE guidelines for reporting observational studies [11] and updated CHEERS guidelines for reporting health economic evaluations [12].

### Setting

The ADAPT study was a cross-sectional study of ADR prevalence in all patients aged ≥ 65 years admitted acutely to a large tertiary referral hospital in Ireland over an eight-month period (November 2016 - June 2017). A subset of those with ADRs and without ADRs (non-ADR) from this initial cross-sectional study were enrolled into a prospective cohort study of patient-reported outcomes and costs associated with ADR-related hospital admissions.

There were 3091 patients initially screened for a suspected ADR-related hospital admission within the first 36 hours of admission by the research team using a previously validated screening process [9]. The Hallas criteria were used to categorise the avoidability/preventability of an ADR [13]. ADR severity was classified using the Hartwig severity assessment scale [14].

The screening approach incorporated a multifaceted review of each hospital admission to assess the likelihood of the ADR being a reason for admission (cause of admission or contributing to admission) in the context of the medications used, clinical conditions, medical history, comorbidities and investigations. In total 361 (11.7%) patients were determined to have an ADR-related admission. A random sample of patients, who were determined not to have a suspected ADR, were assigned to a non-ADR control group for comparative purposes (n = 437).

### Participants

Patients with an ADR-related hospital admission and the non-ADR control group were invited to complete a baseline questionnaire during their hospital stay measuring their health service use, health related quality of life (HRQOL) and medication adherence for the three months prior to hospital admission. Three months post-discharge they were asked to complete a follow-up questionnaire including the same measures. For patients temporarily unable to provide informed consent due to illness severity, their next of kin/carer provided assent to take part in the study and completed the questionnaires on their behalf. Lost to follow-up was defined as non-completion or non-return of the follow-up questionnaire by participants or their proxy.

### Perspective

The perspective of the cost analysis was that of the Irish Health Service Executive (HSE). Publicly financed resources provided within three months before and after discharge were included. This included both accident and emergency, inpatient and outpatient hospital services, GP services, pharmaceuticals, other primary care services, home help and care in nursing homes.

### Data – Sociodemographic and clinical variables

Age at admission, gender (male/female), medical card status (eligibility allows access to medical services, prescription medicines and hospital care for free; Yes/No), smoking status (non-smoker, current smoker, previous smoker), alcohol use (Yes/No), comorbidities and number of medications were extracted from each patient’s medical record. Comorbidity was measured using the Charlson co-morbidity score [15] and polypharmacy was defined as greater than 5 medications and excessive polypharmacy as greater than 10 [16]. Patients additionally self-reported their marital status (no partner, married/partner), level of education (primary, secondary, third level), health insurance status (Yes/No) and living arrangements (with family/relatives/others, live alone, sheltered accommodation) in the three month follow-up questionnaire.

### Data – Health service use

Health service use was measured for all patients with an ADR-related hospital admission and control patients using their medical record and by self-report per their baseline and follow-up questionnaires three months post-discharge. The length of hospital stay (discharge date - admission date) was recorded from their hospital record. The baseline and follow-up questionnaire completed by the individual or proxy measured the utilisation of a total of 25 healthcare items three months pre-admission (baseline) and three months post-discharge (follow-up). This included the number of: (i) GP visits; (ii) out of hours GP services; (iii) hospital visits, A&E visits, hospital inpatient (including duration of stay) and outpatient visits; (iv) use of therapies (e.g. physiotherapy, occupational therapy); (v) use of services (e.g. dietician, optician, pharmacy); (vi) public health nurse and; (vii) use of day care centres and respite care.

### Unit costs

The unit costs for healthcare resources were derived at 2021 Euro (€) price level using HIQA guidelines [10]. For hospital services and GP consultations, we used the national unit costs reported by the HSE Healthcare Pricing Office. For other healthcare services, we made assumptions of the staff salary with addition of 29% add-on costs based in national data for mid-point annual salaries for different staff categories. We assumed 1677 annual working hours (43 weeks at 39 h per week). In addition, based on expert assessment we assumed specific mean durations of all face-to-face contacts with addition of other contact-related time use per contact. We added 40% overhead costs to the direct staff costs. Further, we made explicit assumptions of the cost of transport and use of technologies and facilities as shown in Tables S1a and S1b.

### Statistical analysis

We compared descriptive sociodemographic and clinical variables for the different cohorts (ADR screening, baseline questionnaire, 3-month follow-up questionnaire) within the study population. Descriptive analyses for length of hospital stay are provided as mean and standard deviation (SD). The main analysis was conducted using the ADR-related hospital admission cohort and the non-ADR related hospital admission cohort for individuals who consented to take part and returned both the baseline and 3-month follow-up survey. The main analysis involved comparing the costs associated with healthcare utilisation at baseline and at follow-using the completed surveys. We used Chi-squared and Fisher exact tests to explore statistical significance in group differences for the sociodemographic and clinical categorical variables and an independent samples t-test for comparison of average length of stay between the ADR and non-ADR groups.

For the included pre and post-discharge resource items, we report the proportion of users of each service by the ADR and non-ADR cohorts at baseline and follow-up, and used unadjusted logistic regression to estimate odds ratios (OR) and 95% confidence intervals (CI) for variation in proportion of users at baseline and follow-up, and difference-in-difference odds ratio. We multiplied the number of contacts and the unit cost for each resource item, and summed over all items, and report the mean cost at baseline and follow-up for all individuals in the ADR and non-ADR groups. Generalised linear models with log-link function and gamma distribution were used to estimate mean cost difference-in-difference with and without adjustment for patient characteristics. We interpret these difference-in-difference estimates as the mean incremental cost at follow-up compared to baseline (pre-admission) for the ADR cohort in comparison with the non-ADR cohort.

For the presentation in the main text, we have grouped the resource items at baseline and follow-up into seven cost categories defined by service providers including accident and emergency (A and E) services, hospital services, GP services, pharmacy, other primary care services, home help and nursing, and long-term care services.

We further estimated the difference-in-difference costs for subgroups of the ADR-cohort which had definitely preventable, possible preventable and unavoidable adverse events, and by subgroups with moderate severe and severe ADR. As sample size for these subgroup analyses was limited, the estimation procedure using the specified generalised linear model did not converge, so we estimated these difference-in-difference estimates for aggregated costs using unadjusted ordinary least squares (OLS) regression. All data processing was done with Stata (v17) and significance levels were set at p < 0.05.

## Results

**Fig. 1:**
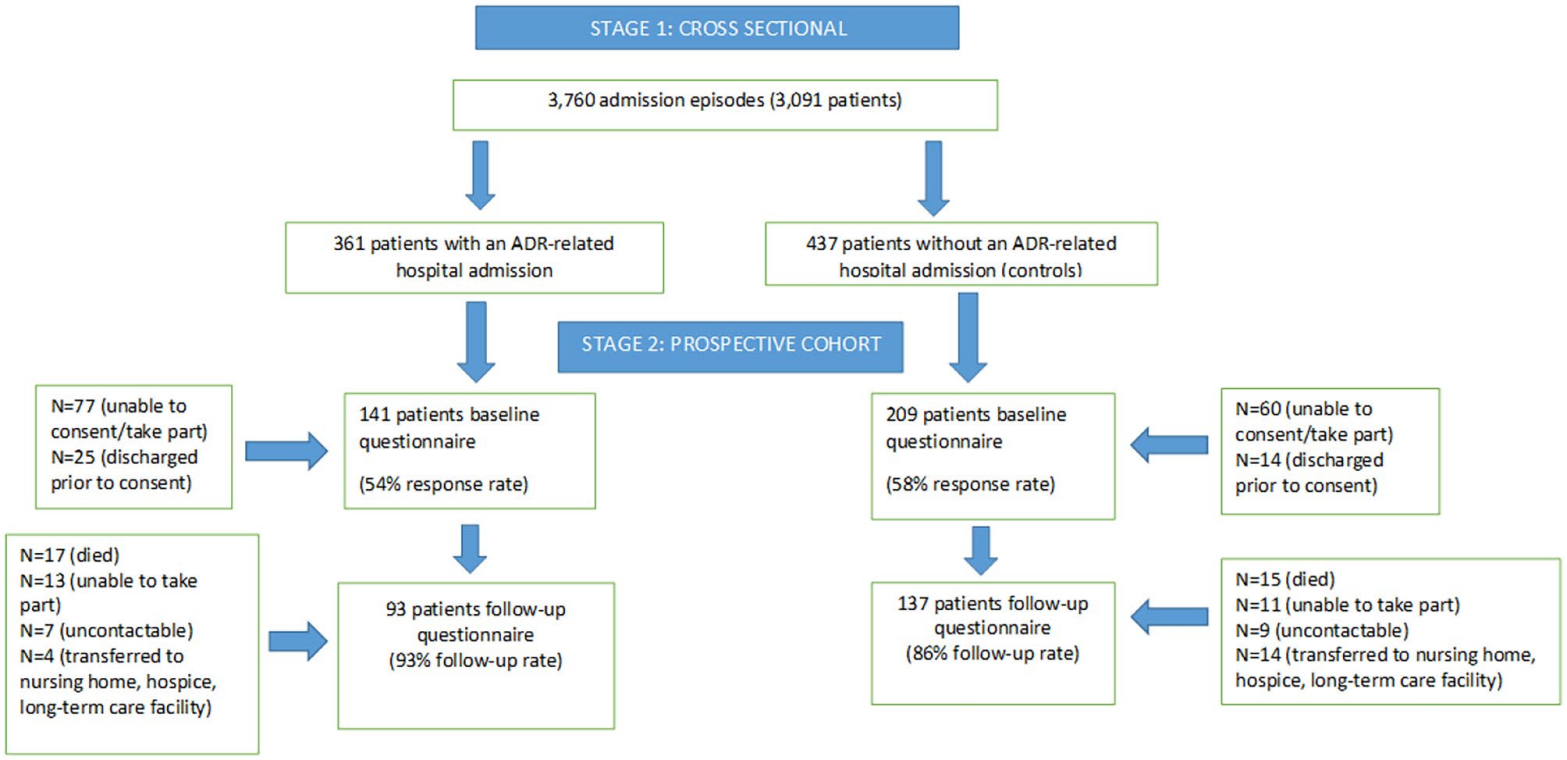
Flow chart of participants into ADR and non-ADR hospital admission cohort study and follow-up at three months post-discharge. Footnote: Unable to take part refers to people who lacked the capacity to consent at baseline or no longer had the capacity to take part in the study at follow-up and had no available proxy (either no family or no contactable family). People who lacked the capacity to consent included those were unconscious or had dementia or were deemed too unwell clinically to be able to participate

There were 350 patients or proxy (carers) who consented to take part in the study at baseline which included 141 with an ADR-related hospital admission and 209 without an ADR-related hospital admission (see Fig. 1). At the 3-month follow-up, a total of 230 patients or proxy (carer) responded to the questionnaire, 93 (9.3% lost to follow-up) in the ADR-related hospital admission group and 137 (8.4% lost to follow-up) in the group without an ADR (Fig. 1). Appendix Table S2 provides further details on characteristics between the screened, baseline and 3-month follow-up populations.

Table 1 presents the sociodemographic and clinical characteristics of those with an ADR-related hospital admission and those without an ADR admission at 3-month follow-up. The data shows similar sociodemographic and clinical characteristics between the two cohorts. A statistically significant higher proportion of patients with an ADR-related hospital admission had third level education, consumed alcohol and were non-smokers (p< 0.05). Of the 93 ADR admissions with follow-up data, 16% were identified as definitely preventable, 61% as possibly preventable and 23% unavoidable. 91% of the ADRs were categorised as moderate severe and 9% as severe.

**Table 1:** Sociodemographic and clinical characteristics of included patients with an ADR-related hospital admission and non-ADR admission at 3-month follow-up

	Non-ADR (total n = 137) n (%)	ADR-related (total n = 93) n (%)	p-value
**Age group**			0.26
< = 69 years	7 (5.1)	11 (11.8)	
70–79 years	58 (42.3)	40 (43.0)	
80–89 years	54 (39.4)	30 (32.3)	
> = 90 years	18 (13.1)	12 (12.9)	
**Gender**			0.17
Male	64 (46.7)	52 (55.9)	
Female	73 (53.3)	41 (44.1)	
**Medical card**			0.41
No	28 (20.4)	15 (16.1)	
Yes	109 (79.6)	78 (83.9)	
**Health insurance**			0.79
No	80 (62.5)	51 (60.7)	
Yes	48 (37.5)	33 (39.2)	
**Marital status**			0.77
No partner	61 (44.9)	75 (55.2)	
Married/partner	39 (42.7)	52 (57.1)	
**Education**			0.03
Primary	70 (54.7)	34 (37.8)	
Secondary	42 (32.8)	36 (40.0)	
Third level	16 (12.5)	20 (22.2)	
**Living arrangements**			0.50
With family/relatives/others	86 (65.6)	62 (67.4)	
Live alone	36 (27.5)	27 (29.4)	
Sheltered accommodation	9 (6.9)	3 (3.4)	
**Alcohol use**			< 0.01
Yes	55 (41.3)	56 (60.2)	
No	78 (58.7)	37 (32.1)	
**Smoking status**			0.02
Non-smoker	49 (36.8)	51 (54.8)	
Current smoker	9 (6.8)	3 (3.23)	
Prev. smoker	75 (56.4)	39 (41.4)	
**Charlson score**			0.54
0	25 (18.2)	16 (17.2)	
1–3	77 (56.2)	52 (55.9)	
4–5	22 (16.1)	11 (11.8)	
6 +	13 (9.5)	14 (15.1)	
**Polypharmacy**			0.45
Non-polypharmacy (≤ 4 drugs)	18 (13.1)	12 (12.9)	
Polypharmacy (5–9 drugs)	71 (51.8)	41 (44.1)	
Excessive polypharmacy (≥ 10 drugs)	48 (35.0)	40 (43.0)	
**ADR avoidability**			
Definitely avoidable		15 (16.1)	
Possibly avoidable		57 (61.3)	
Unavoidable		21 (22.6)	
**ADR severity**			
Moderate		85 (91.4)	
Severe		8 (8.6)	

P-values are based on Chi-squared or Fisher test; Missing data−N=18 health insurance, N=3 marital status, N=12 education level, N=7 living arrangements, N=4 smoking status and alcohol use

The average LOS of the initial hospital admission in the ADR group was 10.8 days (SD = 11.9) and in the non-ADR group 11.2 (SD = 13.4). The mean difference of -0.4 days was not statistically significant (p = 0.85). The average cost associated with the hospital stay was €9538 (SD €10442) in the ADR group and €9828 (SD €11770) in the non-ADR group, with a small difference of -€290 between the ADR and non-ADR group. Admissions for patients with severe ADRs were associated with higher average costs (€9806 (SD €7193)) compared to admissions for patients with moderate severity ADRs (€9516 (SD €10693)). Admissions for possible preventable ADR was associated with higher average costs (€11147 (SD €12356)) compared to admissions with definitely preventable ADR (€7275 (SD €6110)) and unavoidable ADR (€6789 (SD €5361)).

Table 2 presents the main analysis of mean costs of the healthcare provided by different providers at baseline and follow-up for the ADR and non-ADR cohorts and the incremental costs (difference-in-difference) associated with an ADR-induced hospitalisation. The unadjusted mean incremental 3-month costs of an ADR-related hospital admission was estimated at €1427 (95% CI: -€917 to €3771) and the adjusted incremental cost at €2047 (95% CI: -€889 to €4983). The largest healthcare costs pre and post-hospitalisation were associated with accident and emergency department attendance, nursing home costs, and hospital services for the latter estimate.

**Table 2:** Differences in cost of healthcare services by ADR and non-ADR patients (2021-€)

**Healthcare services**	**Observed cost (€), mean (SD)**				**Incremental mean ADR cost (€, 95% CI)**	
	**ADR**	**Non-ADR**	**ADR**	**Non-ADR**		
	**Baseline**	**Baseline**	**Follow-up**	**Follow-up**	**Only follow-up**	**Adjusted difference-in-difference**
A and E services	1711 (6070)	1057 (2389)	1745 (4493)	1926 (3582)	181 (-917;1280)	835 (-870;2540)
Hospital services	856 (2624)	993 (3701)	1621 (6443)	1997 (5609)	376 (-1245;1996)	239 (-1582;2060)
GP services	180 (169)	154 (153)	149 (151)	152 (155)	3 (-38;43)	29 (-31;88)
Pharmaceutical	25 (32)	25 (33)	4 (18)	5 (19)	1 (-4;6)	0 (-10;10)
Other primary care	258 (989)	122 (260)	255 (703)	268 (538)	13 (-157;183)	149 (-118;416)
Home help	380 (1782)	113 (318)	333 (1163)	252 (493)	-81 (-333;171)	186 (-254;626)
Nursing home	349 (2828)	675 (3616)	41 (337)	977 (7202)	935 (-278;2148)	609 (-851;2069)
**Total healthcare services**	**3760 (7318)**	**3140 (5727)**	**4149 (7901)**	**5576 (10,098)**	**1427 (-917;3771)**	**2047 (-889;4983)**

Incremental mean ADR costs for follow−up are estimated with OLS regression; Difference−in−difference estimation adjusted for mean cost difference in baseline and mean cost difference at follow−up. Adjustment included the following covariates: gender, age group, medical card, Charlson score, smoking and alcohol status

Table 3 provides estimates of the incremental costs associated with ADR-related hospital admissions and by preventability and severity of the ADR. The overall estimate of costs per ADR associated with definitely preventable ADR was €1648 (95% CI: -€4310 to €7605), with possibly preventable €2259 (95% CI: -€1194 to €5712) and with unavoidable €1757 (95% CI: -€3377 to €6890). For those with moderate severe ADR the costs were estimated at €1922 (95% CI: –€1088 to €4932) and €3580 with severe ADR (95% CI: -€4898 to €12058).

**Table 3:** Differences-in-difference costs of healthcare services associated with preventable ADR and severity of ADRs

**Healthcare services**	**Difference in difference costs related to ADR (2021-€) and 95% CI**				
	**Def. preventable**	**Pos. preventable**	**Unavoidable**	**Moderate severity ADR**	**Severe ADR**
	n = 15	n = 57	n = 21	n = 86	n = 7
**A and E services**	347 (-3110;3805)	1098 (-906;3102)	471 (-2508;3450)	823 (-922;2569)	982 (-3934;5899)
**Hospital services**	729 (-2923;4380)	-169 (-2285;1947)	997 (-2150;4143)	110 (-1749;1968)	1824 (-3411;7059)
**GP services**	-45 (-164;74)	20 (-49;89)	104 (2;207)	36 (-24;96)	-63 (-233;107)
**Pharmaceutical**	-12 (-32;8)	-0 (-12;11)	10 (-7;28)	-2 (-12;8)	30 (1;58)
**Other primary care**	154 (-388;696)	83 (-231;397)	325 (-142;792)	143 (-131;417)	229 (-543;1000)
**Home help**	65 (-839;969)	245 (-280;769)	114 (-665;893)	179 (-277;636)	271 (-1015;1557)
**Nursing home**	409 (-2573;3392)	983 (-746;2711)	-264 (-2834;2306)	633 (-875;2142)	307 (-3941;4556)
**Total healthcare services**	1648 (-4310;7605)	2259 (-1194;5712)	1757 (-3377;6890)	1922 (-1088;4932)	3580 (-4898;12058)

Difference-in-difference ADR costs estimated with OLS regression

## Discussion

Our study findings suggest a modest difference in costs associated with an ADR-related hospitalisation and post-discharge compared to non-ADR hospitalisation with an average decrease of €290 in the costs association with the initial hospital admission and an average increase in pre and post-hospitalisation costs of €2050 per individual identified with an ADR compared to non-ADR. The majority of the differences in additional costs not associated with the index hospital stay were attributed to the increases in nursing home care, increased accident and emergency department visits and hospital services after discharge for their acute episode in the ADR cohort. The average costs associated with ADRs were highest in those with possibly preventable ADRs and those with moderate severity or severe ADRs.

There have been a small number of systematic reviews of studies examining the costs associated with ADRs or adverse drug events (ADEs) [6, 7] which is defined as ‘any injuries resulting from medication use, including physical harm, mental harm, or loss of function’ [17]. The studies within these reviews are not focused specifically on the elderly and are conducted across different countries, settings and populations with different methodological approaches. In particular, a systematic review of thirty-one observational studies of ADEs found that the average “direct costs” in ambulatory care ranged from €702 to €40273, and average in-hospital costs from €943 to €7192 [6]. There were significant methodological differences relating to the design, type of ADEs included and the type and structure of costs, but the costs remain higher across most studies. The studies were presented separately according to whether the ADE led to hospitalisation or occurred during hospitalisation. All ages were included and only one study in the review referred to indirect costs associated with ADEs leading to hospitalisation [18]. In this study the incremental cost per patient with an ADE was €1982. A German study included in the systematic review conducted a micro-costing study of ADEs using a retrospective and medical record–based study [19]. Those hospitalised for ADEs were matched to a non-ADE cohort with associated mean costs of €5113 and €4143 respectively (a difference of €970 ~ 23%). Another review examined observational studies to evaluate the economic impact of preventable ADRs and found the costs due to preventable ADRs in an inpatient setting had a wider range than outpatient setting: a minimum of €2851 to a maximum of €9015 in the inpatient setting compared to a minimum of €174 to a maximum of €8515 in the outpatient setting [7].

One recent study in the UK found that the average costs associated with hospitalisation for medication harm (including ADRs) and healthcare utilisation after 8 weeks post-discharge was approximately £550 [20]. Another study using the US Veterans Health Administration (VHA) database examined the costs of severe ADRs by drug-symptom pairs in 5113 outpatient ADR reports from 4880 veterans [21]. The authors were not able to report on total costs due to spontaneous reporting of ADRs. Of those pairs reported, reflecting more severe ADRs, the average costs were high ranging from US$9930 to US$49258. Another UK study extrapolated the costs of ADRs, based on a one-month study period over which data on ADRs were captured, and applied this to national population figures [22]. A study in 2007 on the costs of emergency department (ED) visits related to ADRs for patients greater than 65 years of age using administrative data in Ontario, Canada found that ADR-related visits were $333 per ED visit and $7528 per hospitalization or an estimated $35.7 million in Canada [23].

*Strengths and Limitations* – Our study provides additional unique features, including the elaborate and careful identification of an ADR on admission, the classification of ADRs using validated criteria, the comprehensive collection of healthcare resource use before and after hospital admission, and robust analysis and reporting of the healthcare resource use and costs. Other strengths are the inclusion of screened ADRs in all those admitted to the tertiary centre and the comparison to a non-ADR population.

However, there are a number of limitations. Comparisons with other studies are difficult due to differences in populations, definitions used, medications included, and cost sources. In addition, our study population were much older and, therefore, at higher risk of ADRs due to underlying comorbidities and polypharmacy. In particular, a high proportion were admitted with cardiovascular medicines and one in four on antidepressants. [9] In addition, there were differences in key disease areas in each cohort, for example lower prevalence of chronic lung disease but higher prevalence of ulcer disease in the ADR cohort, which may also have had an effect on the utilisation of health care resources and costs.

Also, the older aged populations are likely to be associated with other contributory factors such as complex medication regimens, cognition issues, vision deficits, nutrition, mobility/falls and fracture risk, social supports. Therefore, generalisability to other populations and settings may be limited. The sample size in the ADR and non-ADR cohorts was relatively small, and only 66% of the ADR and non-ADR cohorts provided data at the 3-month follow-up. There are also limitations related to estimates of costs which are based on estimated time and units costs available.

ADR-related hospitalisation is a significant burden among the older aged population and often preventable. The costs associated with ADR-hospitalisation are modest, but also depend on the severity of the ADR. However, the cost is not only financial but is often associated with costs both clinically and personally for individuals. Early intervention, where possible, is important to avoid preventable medication harm. Intervention with more targeted policies to reduce ADRs through identification of those at highest risk and more awareness among HCPs and others involved in the care of the older populations will help to reduce costly hospitalisations and avoid increased morbidity and mortality [24]. A recent review of tools to help predict and detect ADR in older aged patients (≥ 60 years) identified eighteen studies using a variety of tools, but no one definitive and validated assessment tool for detecting and predicting ADR in older aged populations [25]. Therefore, more research is required to develop validated tools that can be implemented in clinical practice [26–28]. Empowering individuals and their care-givers through increased health literacy and education may help to reduce ADR-related hospitalisation and associated costs, and improving the transitions of care and pharmacy reviews are alternative interventions to help limit the impact of ADRs [29].

In conclusion, ADR-related hospitalisation and post-discharge care in older individuals results in significant healthcare utilisation and costs. The costs are increased with preventability and severity of the ADR. More research is required to develop validated tools for prevention and early detection of ADRs that can be implemented in clinical practice to avoid unnecessary harm and burden, including economic burden.

## Supplementary Information

Below is the link to the electronic supplementary material.

Supplementary file1 (DOCX 54 KB)

Supplementary file2 (DOCX 53 KB)

## Data Availability

The dataset generated during and/or analysed during the current study are available from the corresponding author on reasonable request.
