# Supplementary material for: Costs associated with adverse drug reactions in an older population admitted to hospital: a prospective cohort study
Source: Eur J Clin Pharmacol. 2023 Aug 24;79(10):1417–24. doi: 10.1007/s00228-023-03552-x (PMC10501947; doi:10.1007/s00228-023-03552-x)
Supplement: Supplementary file 1 — Supplementary file1 (DOCX 54 KB) [file 228_2023_3552_MOESM1_ESM.docx]

**SUPPLEMENTARY APPENDIX**

**Table S1a. Unit cost derived from national charges**

| **Resource** | **Unit cost €** |
| --- | --- |
| Inpatient admissions excl. bed day costs | 2000 |
| Bed days (hotel cost) | 880 |
| GP consultations | 48 |
| GP out of hours consultations | 97 |
| Hosp A&E admissions | 279 |
| Hosp A&E stay | 590 |
| Hosp outpatient visits | 131 |

^Source: Information obtained from the Healthcare Pricing Office^

**Table S1b. Calculation of unit cost based on assumptions related to salary, contact duration, and transports.**

| **Services** | **Annual staff**  **Salary €** | **Duration of face-to-face contact [min]** | **Add-on time for admin. and indirect**  **patient work**  **[min]** | **Transport costs €** | **Costs related to facilities**  **€** | **Estimated unit cost**  **€** |
| --- | --- | --- | --- | --- | --- | --- |
| Public Health Nurse visit | 46857 | 60 | 20 | 50 | 0 | 117 |
| Physiotherapy visit | 34969 | 30 | 15 | 0 | 25 | 53 |
| Occupational.therapy visit | 34969 | 60 | 15 | 0 | 25 | 72 |
| Speech & Lang visit | 34969 | 60 | 10 | 0 | 10 | 54 |
| Dietician visit | 34969 | 90 | 10 | 0 | 10 | 73 |
| Optician Visit | 34969 | 60 | 10 | 0 | 10 | 54 |
| Dental visit | 59270 | 30 | 10 | 0 | 25 | 68 |
| Hearing service visit | 34969 | 60 | 10 | 0 | 10 | 54 |
| Chiropody visit | 34969 | 30 | 10 | 0 | 10 | 35 |
| Psychologist visit | 49585 | 60 | 10 | 0 | 10 | 72 |
| Day care centre visit (da | 27044 | 300 | 0 | 75 | 100 | 321 |
| Respite care visit (days) | 27044 | 300 | 0 | 75 | 100 | 321 |
| Day care visit (days) | 26001 | 150 | 0 | 75 | 100 | 245 |
| Chiropractor visit | 31088 | 60 | 10 | 0 | 10 | 49 |
| Alternative therapy visit | 26001 | 45 | 10 | 0 | 10 | 36 |
| Pharmacist cons. | 32831 | 30 | 10 | 0 | 10 | 34 |
| Home help hours | 26001 | 60 | 20 | 50 | 0 | 87 |
| Private Hope help hours | 26001 | 60 | 20 | 50 | 0 | 87 |

^Note: Hourly salary cost calculated as the annual salary multiplied by 1.29 (add-on staff cost) and by 1.4 (overhead) divided by 43 work weeks € 39 work hours per week. Unit cost calculated as duration of visit + add-on time multiplied by hourly salary cost with addition of transport cost and facilities cost.^

**Table S2. Characteristics of study participants**

|  |  | **Screened** | **No consent** | | **Consented: Baseline data** | | **Consented: Follow-up data** | | **p-value: Consented of Screened** | | **p-value: With follow-up of Consented** | | **p-value: With FU of Consented** | |
| --- | --- | --- | --- | --- | --- | --- | --- | --- | --- | --- | --- | --- | --- | --- |
| **n (%)** |  | 798 (100.0) | 448 (56.1) | | 350 (43.9) | | 230 (28.8) | |  | |  | |  | |
| **Adverse drug reaction, n (%)** | | | |  | |  | | 0.013 | | 0.083 | | 0. 937 | |  |
| Identified with ADR |  | 361 (45.2) | 220 (49.1) | | 141 (40.3) | | 93 (40.4) | |  | |  | |  | |
| Without ADR |  | 437 (54.8) | 228 (50.9) | | 209 (59.7) | | 137 (59.6) | |  | |  | |  | |
| **Gender, n (%)** | | | |  | |  | | 0.120 | | 0.333 | | 0.827 | |  |
| Male |  | 381 (47.7) | 203 (45.3) | | 178 (50.9) | | 116 (50.4) | |  | |  | |  | |
| Female |  | 417 (52.3) | 245 (54.7) | | 172 (49.1) | | 114 (49.6) | |  | |  | |  | |
| **Age group, n (%)** | | | |  | |  | | 0.313 | | 0.157 | | 0.622 | |  |
| <=69 |  | 48 (6.0) | 23 (5.1) | | 25 (7.1) | | 18 (7.8) | |  | |  | |  | |
| 70-79 |  | 309 (38.7) | 166 (37.1) | | 143 (40.9) | | 98 (42.6) | |  | |  | |  | |
| 80-89 |  | 316 (39.6) | 183 (40.8) | | 133 (38.0) | | 84 (36.5) | |  | |  | |  | |
| >=90 |  | 125 (15.7) | 76 (17.0) | | 49 (14.0) | | 30 (13.0) | |  | |  | |  | |
| **Medical card eligibility, n (%)** | | | |  | |  | | <0.001 | | <0.001 | | 0.768 | |  |
| No |  | 515 (64.5) | 448 (100.0) | | 67 (19.1) | | 43 (18.7) | |  | |  | |  | |
| Yes |  | 283 (35.5) | 0 (0.0) | | 283 (80.9) | | 187 (81.3) | |  | |  | |  | |
| **Alcohol, n (%)** | | | |  | |  | | 0.006 | | 0.002 | | 0.092 | |  |
| Yes |  | 306 (40.5) | 150 (36.1) | | 156 (45.9) | | 111 (49.1) | |  | |  | |  | |
| No |  | 450 (59.5) | 266 (63.9) | | 184 (54.1) | | 115 (50.9) | |  | |  | |  | |
| **Smoking status, n (%)** | | | |  | |  | | 0.010 | | 0.010 | | 0.200 | |  |
| Non-smoker |  | 339 (44.8) | 193 (46.4) | | 146 (42.9) | | 100 (44.3) | |  | |  | |  | |
| Current smoker |  | 76 (10.1) | 52 (12.5) | | 24 (7.1) | | 12 (5.3) | |  | |  | |  | |
| Ex-smoker |  | 341 (45.1) | 171 (41.1) | | 170 (50.0) | | 114 (50.4) | |  | |  | |  | |
| **Charlson score, n (%)** | | | |  | |  | | 0.558 | | 0.990 | | 0.287 | |  |
| 0 |  | 138 (17.3) | 85 (19.0) | | 53 (15.2) | | 41 (17.8) | |  | |  | |  | |
| 1-3 |  | 451 (56.6) | 247 (55.1) | | 204 (58.5) | | 129 (56.1) | |  | |  | |  | |
| 4-5 |  | 117 (14.7) | 66 (14.7) | | 51 (14.6) | | 33 (14.4) | |  | |  | |  | |
| 6+ |  | 91 (11.4) | 50 (11.2) | | 41 (11.7) | | 27 (11.7) | |  | |  | |  | |
| **Polypharmacy** |  |  |  | |  | |  | |  | |  | |  | |
| Non-polypharmacy (≤4 drugs) |  | 110 (13.8) | 66 (14.7) | | 44 (12.6) | | 30 (13.0) | | 0.654 | | 0.817 | | 0.564 | |
| Polypharmacy (5–9 drugs) |  | 375 (47.0) | 210 (46.9) | | 165 (47.1) | | 112 (48.7) | |  | |  | |  | |
| Excessive polypharmacy (≥10 drugs) |  | 313 (39.2) | 172 (38.4) | | 141 (40.3) | | 88 (38.3) | |  | |  | |  | |

^N= 42 missing for alcohol and smoking status^

^N=1 missing for Charlson score^

**Table S3. Differences in use of healthcare services by Non-ADR and ADR patients**

| **Healthcare services** | **Baseline** | | **Follow-up** | | **Odds-ratio (95% CI)** |  |  |
| --- | --- | --- | --- | --- | --- | --- | --- |
|  | **User %** | | **User %** | | **Baseline** | **Follow-up** | **Diff-in-diff** |
|  | Non-ADR | ADR | Non-ADR | ADR |  |  |  |
| GP consultations | 85.4 | 82.8 | 76.6 | 75.3 | 0.82 (0.40 to 1.69) | 0.93 (0.50 to 1.72) | 1.13 (0.43 to 2.97) |
| GP out of hours cons | 18.2 | 12.9 | 13.1 | 15.1 | 0.66 (0.31 to 1.40) | 1.17 (0.55 to 2.49) | 1.81 (0.61 to 5.34) |
| A&E visits | 67.9 | 64.5 | 40.9 | 48.4 | 0.86 (0.49 to 1.50) | 1.36 (0.80 to 2.30) | 1.60 (0.73 to 3.48) |
| A&E overnight stay | 23.4 | 20.4 | 19.7 | 33.3 | 0.84 (0.44 to 1.60) | 2.04 (1.12 to 3.72) | 2.49 (1.02 to 6.07) |
| Inpatient admissions | 13.9 | 11.8 | 9.5 | 15.1 | 0.83 (0.38 to 1.84) | 1.69 (0.76 to 3.78) | 2.11 (0.66 to 6.76) |
| Inpatient bed days | 2.9 | 6.5 | 8.0 | 12.9 | 2.29 (0.63 to 8.36) | 1.70 (0.71 to 4.03) | 0.74 (0.15 to 3.55) |
| Outpatient visits | 45.3 | 41.9 | 40.9 | 57.0 | 0.87 (0.51 to 1.49) | 1.92 (1.12 to 3.27) | 2.32 (1.06 to 5.07) |
| Public nurse visits | 19.0 | 19.4 | 20.4 | 30.1 | 1.02 (0.53 to 2.00) | 1.68 (0.91 to 3.08) | 1.67 (0.67 to 4.21) |
| Physiotherapist visits | 18.2 | 7.5 | 17.5 | 11.8 | 0.36 (0.15 to 0.88) | 0.63 (0.29 to 1.36) | 1.77 (0.54 to 5.86) |
| Occupation therapist visits | 10.9 | 7.5 | 13.9 | 9.7 | 0.66 (0.26 to 1.69) | 0.67 (0.29 to 1.54) | 1.00 (0.28 to 3.66) |
| Speech therapist visits | 4.4 | 0.0 | 2.2 | - | - | 0.98 (0.16 to 5.99) | 1.00 ( . to .) |
| Dietitian visits | 5.1 | 1.1 | 5.1 | 6.5 | 0.20 (0.02 to 1.67) | 1.28 (0.42 to 3.94) | 6.50 (0.59 to 72.07) |
| Optician visits | 14.6 | 11.8 | 13.1 | 14.0 | 0.78 (0.36 to 1.73) | 1.07 (0.50 to 2.31) | 1.38 (0.45 to 4.25) |
| Dentist visits | 13.9 | 9.7 | 9.5 | 8.6 | 0.67 (0.29 to 1.54) | 0.90 (0.36 to 2.26) | 1.37 (0.39 to 4.90) |
| Hearing services visits | 8.8 | 6.5 | 2.9 | 5.4 | 0.72 (0.26 to 1.99) | 1.89 (0.49 to 7.23) | 2.80 (0.49 to 15.85) |
| Chiropody visits | 24.8 | 20.4 | 22.6 | 21.5 | 0.78 (0.41 to 1.47) | 0.94 (0.50 to 1.77) | 1.22 (0.48 to 3.08) |
| Psychologist visits | 2.9 | 1.1 | 1.5 | 1.1 | 0.36 (0.04 to 3.29) | 0.73 (0.07 to 8.21) | 2.18 (0.08 to 61.46) |
| Day care visits | 2.9 | 3.2 | 1.5 | 6.5 | 1.11 (0.24 to 5.07) | 4.66 (0.92 to 23.59) | 4.60 (0.46 to 45.88) |
| Respite care visits | 3.6 | 1.1 | 1.5 | 5.4 | 0.29 (0.03 to 2.50) | 3.84 (0.73 to 20.20) | 17.46 (1.00 to 304.79) |
| Respite care stay days | 0.7 | 0.0 | 0.7 | - | - | 1.48 (0.09 to 23.93) | 1.00 ( . to .) |
| Chiropractor visits | 1.5 | 2.2 | 0.7 | 1.1 | 1.48 (0.21 to 10.72) | 1.48 (0.09 to 23.93) | 0.99 (0.03 to 30.99) |
| Alternative practice visits | 2.2 | 1.1 | - | - | - | 1.00 ( . to .) | 1.00 ( . to .) |
| Pharmacist visits | 38.7 | 38.7 | 7.3 | 7.5 | 1.00 (0.58 to 1.72) | 1.03 (0.38 to 2.82) | 1.03 (0.33 to 3.26) |
| Public home help | 24.1 | 18.3 | 25.5 | 30.1 | 0.70 (0.37 to 1.36) | 1.26 (0.70 to 2.26) | 1.88 (0.75 to 4.74) |
| Private home help | 3.6 | 2.2 | 4.4 | 3.2 | 0.58 (0.11 to 3.06) | 0.73 (0.18 to 2.99) | 1.25 (0.13 to 11.64) |

**Table S4. Differences in cost of healthcare services by non-ADR and ADR patients (2021-€)**

| **Healthcare services** | **Observed cost, mean (sd)** | | | | **Incremental cost** | |
| --- | --- | --- | --- | --- | --- | --- |
|  | **ADR** | **Non-ADR** | **ADR** | **Non-ADR** | **Only follow-up** | **Adj. diff-in-diff** |
|  | **Baseline** | **Baseline** | **Follow-up** | **Follow-up** | **€ mean (95%CI)** | **€ mean (95%CI)** |
| Cost of GP consultations | 156 (138) | 138 (137) | 130 (126) | 128 (122) | -1 (-34;32) | 17 (-32;66) |
| Cost of GP out of hours cons | 24 (59) | 59 (53) | 20 (63) | 24 (82) | 4 (-15;23) | 12 (-13;36) |
| Cost of A&E visits | 277 (305) | 305 (248) | 216 (353) | 213 (295) | -3 (-90;85) | 10 (-105;125) |
| Cost of A&E overnight stay | 1434 (6004) | 6004 (2279) | 1529 (4361) | 1713 (3461) | 184 (-881;1249) | 825 (-843;2493) |
| Cost of Inpatient admissions | 540 (1724) | 1724 (991) | 336 (1126) | 473 (1426) | 137 (-195;470) | 355 (-154;864) |
| Cost of Inpatient bed days | 122 (1073) | 1073 (2863) | 1169 (6078) | 1344 (5016) | 175 (-1327;1677) | -243 (-1843;1358) |
| Cost of Outpatient visits | 194 (1013) | 1013 (263) | 117 (209) | 180 (221) | 64 (7;120) | 127 (-92;346) |
| Cost of Public nurse visits | 159 (975) | 975 (246) | 158 (607) | 177 (489) | 19 (-129;168) | 108 (-143;359) |
| Cost of Physiotherapist visits | 36 (144) | 144 (36) | 21 (55) | 14 (46) | -6 (-20;7) | 21 (-12;54) |
| Cost of Occupation therapist visits | 14 (51) | 51 (37) | 23 (90) | 29 (122) | 6 (-22;33) | 11 (-19;41) |
| Cost of Speech therapist visits | 3 (17) | 17 (0) | 3 (28) | 3 (23) | -0 (-7;7) | 3 (-5;11) |
| Cost of Dietitian visits | 4 (19) | 19 (8) | 7 (41) | 7 (34) | 0 (-10;10) | 4 (-7;15) |
| Cost of Optician visits | 9 (22) | 22 (22) | 12 (40) | 11 (31) | -1 (-11;9) | -0 (-11;11) |
| Cost of Dentist visits | 10 (25) | 25 (29) | 13 (61) | 10 (40) | -3 (-17;11) | -2 (-17;14) |
| Cost of Hearing services visits | 5 (15) | 15 (13) | 2 (14) | 3 (16) | 1 (-3;5) | 2 (-3;8) |
| Cost of Chiropody visits | 10 (18) | 18 (23) | 10 (20) | 10 (20) | -0 (-6;5) | -0 (-8;7) |
| Cost of Psychologist visits | 5 (31) | 31 (15) | 4 (39) | 2 (22) | -2 (-11;7) | 1 (-10;12) |
| Cost of Day care visits | 84 (566) | 566 (2747) | 25 (254) | 234 (1573) | 209 (-60;479) | -133 (-673;407) |
| Cost of Respite care visits | 232 (2472) | 2472 (2397) | 7 (61) | 625 (5593) | 618 (-323;1558) | 601 (-535;1738) |
| Cost of Respite care stay days | 33 (384) | 384 (0) | 9 (110) | 117 (1132) | 108 (-84;300) | 141 (-68;349) |
| Cost of Chiropractor visits | 1 (6) | 6 (11) | 0 (4) | 1 (5) | 0 (-1;1) | -1 (-3;2) |
| Cost of Alternative pract visits | 4 (38) | 38 (7) | 1 (7) | 0 (0) | -1 (-3;0) | 2 (-6;10) |
| Cost of Pharmacist visits | 25 (32) | 32 (33) | 4 (18) | 5 (19) | 1 (-4;6) | 0 (-10;10) |
| Cost of Public home help | 271 (1295) | 1295 (318) | 198 (456) | 247 (493) | 49 (-76;174) | 209 (-89;506) |
| Cost of Private home help | 110 (1249) | 1249 (20) | 135 (1093) | 5 (27) | -130 (-354;94) | -23 (-357;311) |
| Total cost | 3760 (7318) | 7318 (5727) | 4149 (7901) | 5576 (10098) | 1427 (-917;3771) | 2047 (-889;4983) |

Note: Incremental ADR costs estimated using OLS-regression. Difference in difference estimation adjusts for baseline difference, and difference over time for the non-ADR group. Adjustment for gender, age group, medical card, Charlson score, smoking and alcohol status.
